# Comprehensive Amelioration of Metabolic Dysfunction through Administration of *Lactiplantibacillus plantarum* APsulloc 331261 (GTB1™) in High-Fat-Diet-Fed Mice

**DOI:** 10.3390/foods13142227

**Published:** 2024-07-16

**Authors:** Bobae Kim, Yuri Lee, Chungho Lee, Eun Sung Jung, Hyeji Kang, Wilhelm H. Holzapfel

**Affiliations:** 1 Basic Research Center, HEM Pharma Inc., Pohang 37554, Republic of Korea; kbb0427@naver.com (B.K.); youri4@naver.com (Y.L.); chungholee45@gmail.com (C.L.); 2 Department of Advanced Convergence, Handong Global University, Pohang 37554, Republic of Korea; 3 Multi-Omics Center, HEM Pharma Inc., Suwon 16229, Republic of Korea; esjung@hempharma.bio; 4 Global Green Research Institute, Handong Global University, Pohang 37554, Republic of Korea

**Keywords:** *Lactobacillus plantarum* APsulloc 331261 (GTB1™), obesity, metabolic dysfunction, gut microbiota modulation, short-chain fatty acids (SCFAs), peroxisome proliferator-activated receptors (PPARs)

## Abstract

The beneficial effects of probiotics for the improvement of metabolic disorders have been studied intensively; however, these effects are evident in a probiotic strain-specific and disease-specific manner. Thus, it is still essential to evaluate the efficacy of each strain against a target disease. Here, we present an anti-obese and anti-diabetic probiotic strain, *Lactiplantibacillus plantarum* APsulloc331261 (GTB1™), which was isolated from green tea and tested for safety previously. In high-fat-diet-induced obese mice, GTB1™ exerted multiple beneficial effects, including significant reductions in adiposity, glucose intolerance, and dyslipidemia, which were further supported by improvements in levels of circulating hormones and adipokines. Lipid metabolism in adipose tissues was restored through the activation of PPAR/PGC1α signaling by GTB1™ treatment, which was facilitated by intestinal microbiota composition changes and short-chain fatty acid production. Our findings provide evidence to suggest that GTB1™ is a potential candidate for probiotic supplementation for comprehensive improvement in metabolic disorders.

## 1. Introduction

Obesity is a chronic, complex disease where excessive ectopic lipid accumulation leads to the development of metabolic syndrome, in which a group of metabolic dysfunctions coexist, such as glucose intolerance, dyslipidemia, and non-alcoholic fatty liver disease (NAFLD) [1]. The prevalence of obesity has more than doubled since 1990, and 1 in 8 people were living with obesity in 2022 [2]. Resolving obesity status is a critical issue because it is correlated with an increased risk for various metabolic, cardiovascular, and skeletal co-morbidities, which are deeply associated with the world’s leading cause of mortality. Obesity treatment guidelines point out that weight management should include multiple approaches at the same time, including lifestyle modifications, medication, and/or bariatric surgery [3,4]. The development of anti-obesity medication is highly challenging because it requires both constant efficacy and long-term consumption safety [5]. In particular, several serious adverse effects such as paradoxically increased cardiovascular disease rate, drug abuse/dependency, and cancer, have been reported for previously developed drugs [6]. Therefore, there is a high demand for the development of novel obesity treatments.

Over the last two decades, the intestinal microbiota has become recognized as one of the main regulators of the host metabolism, and the changes in its composition could contribute to the pathogenesis of obesity and related diseases [7]. From this perspective, there have been constant efforts in both animal studies and clinical trials to adapt various probiotic strains for intervention in intestinal microbiota dysbiosis and metabolic dysfunctions, particularly those of *Lactobacillus* and *Bifidobacterium* [8]. In these previous studies, the probiotics were described as ameliorating dysregulated energy metabolism, not only by improving the intestinal microbiota, but also by enhancing metabolite production and altering host immune status [9,10,11]. However, probiotics being strain-specific and disease-specific make it difficult to select the appropriate strain from the variety of origins [12]. Thus, it is still essential to investigate the efficacy of one specific probiotic strain against a target disease and to discover the underlying mechanism behind the beneficial effects.

Green tea is one of the major classifications of tea (*Camellia sinensis*) that belongs to the non-fermented tea class [13]. The health-promoting effects of green tea have been constantly reported and include a wide range of benefits, including against obesity, cardiovascular diseases, cancer, arthritis, and neurodegenerative diseases; these effects are mostly related to its abundant bioactive components, such as polyphenols [14]. Interestingly, these phenolic compounds from green tea extract were reported to selectively inhibit the growth of pathogenic bacteria, whereas the species *Lactiplantibacillus (Lp.) plantarum* was able to metabolize phenolic acids and tannins and potentiate their growth [15,16]. In this study, we present an anti-obese and anti-diabetic probiotic strain, *Lp. plantarum* APsulloc331261 (GTB1™), which was isolated from green tea and tested for safety previously [17], in a high-fat-diet (HFD)-induced obese mouse model. GTB1™ exerted multiple beneficial effects, including significant reductions in adiposity, glucose intolerance, and dyslipidemia, which were further supported by modulations in circulating hormones and adipokines, lipid metabolism in adipose tissues, and the intestinal microbiota composition. Our findings provide evidence to suggest that GTB1™ is a potential candidate for probiotic supplementation for comprehensive improvements in metabolic disorders.

## 2. Materials and Methods

### 2.1. Preparation of Bacterial Strain

*Lp. plantarum* APsulloc 331261 (GTB1™) was kindly provided by Amorepacific Corp. (KCCM11179P; Yongin, Republic of Korea) and was firstly isolated from green tea (Dolsongi tea field, Jeju Island, Republic of Korea) [17]. The strain was grown in MRS broth (Difco Laboratories Inc., Franklin Lakes, NJ, USA) at 37 °C for 16 h, collected by centrifugation at 3000× *g* for 15 min at 4 °C, washed twice with sterile phosphate-buffered saline (PBS), and then resuspended in PBS at the concentration of 1 × 10^8^ or 1 × 10^9^ CFU/200 µL PBS for oral administration to mice. 

### 2.2. Animal Experiment

Five-week-old C57BL/6J male mice provided by Saeronbio Inc. (Seongnam, Republic of Korea) were kept in a temperature- and humidity-controlled environment (22 ± 1 °C and 55 ± 10%) with a 12 h light/dark cycle. Mice were acclimatized to the environment for 1 week, fed with normal chow diet (Purina, Chicago, IL, USA), and then divided into five groups (*n* = 9 per group): low-fat-diet (LF)-fed, PBS-treated control, high-fat-diet (HF)-fed, PBS-treated control, HF-fed, metformin (300 mg/kgBW; D150959, Sigma-Aldrich, Saint Louis, MO, USA)-treated, HF-fed, low-dose (1 × 10^8^ CFU/day/mouse) *Lp. plantarum* APsulloc 331261 (GTB1™)-treated, and HF-fed, high-dose (1 × 10^9^ CFU/day/mouse) *Lp. plantarum* APsulloc 331261 (GTB1™)-treated. Each group was fed with LF (10%kcal from fat, D12450J, Research Diets Inc., New Brunswick, NJ, USA) or HF (60%kcal from fat, D12492, Research Diets Inc.) for 1 week, and then oral gavaged with 200 µL PBS or a daily dose probiotic for an additional 14 weeks with each designated diet being fed. 

At the end of the experimental period, mice were starved for 4 h and euthanized and their tissues were collected. To collect plasma samples, 700 μL of blood samples were drawn from the heart, transferred to a BD Microtainer^®^ plasma separation tube (#365985, Becton, Dickinson and Company, Franklin Lakes, NJ, USA), and centrifuged at 1500× *g* for 15 min at room temperature. Tissues samples from subcutaneous adipose tissue (SAT), epididymal adipose tissue (EAT), mesenteric adipose tissue (MAT), the liver, quadriceps, brown adipose tissue (BAT), and cecum were harvested and stored at −70 °C for further analyses. All animal experiment procedures were approved by the Committee on the Ethics of Animal Experiments of the Handong Global University (Permit number: 20190328-011).

### 2.3. Glucose Tolerance Test and Insulin Tolerance Test 

A glucose tolerance test and insulin tolerance test were carried out with mice at 13 weeks of treatment. For the glucose tolerance test, mice were fasted for 6 h and then intraperitoneally injected with glucose (2 g/kgBW). For the insulin tolerance test, mice were fasted for 4 h and received an intraperitoneal injection of 0.75 U/kgBW insulin. Blood samples were collected by tail-bleeding, and the level of blood glucose was measured at 0, 15, 30, 60, 90, and 120 min after injection by GlucoDr auto AGM-4000 (Allmedicus Inc., Anyang, Republic of Korea).

### 2.4. Plasma Analyses

Measurements of insulin (Morinaga Institute of Biological Science Inc., Yokohama, Japan), leptin (Elabscience Biotechnology Inc., Houston, TX, USA), and adiponectin (R&D systems, Minneapolis, MN, USA) in plasma were performed with commercial ELISA kits according to the manufacturer’s instructions. Plasma levels of alanine transaminase (ALT), aspartate aminotransferase (AST), triglyceride (TG), total cholesterol (CHOL), high-density lipoprotein cholesterol (HDL-C), and low-density lipoprotein cholesterol (LDL-C) were measured using an automated biochemistry analyzer (BS-390, Mindray Bio-medical Electronics Co., Ltd., Shenzhen, China).

### 2.5. Histological Analysis

Tissue samples were fixed in 10% *v*/*v* formalin/PBS, paraffin-embedded, and then stained with hematoxylin and eosin (H&E). Images were obtained using a light microscope (Carl Zeiss Microscopy GmbH, Göttingen, Germany) at a magnification of ×100. The adipocyte size and number were quantified using ImageJ software version 1.54 (National Institutes of Health, Bethesda, MD, USA) with the Adiposoft plug-in according to the developer’s instructions [18].

### 2.6. Hepatic Triglyceride (TG) Quantification

Triglyceride (TG) in the liver was extracted and quantified as described previously [19]. Briefly, the liver tissue was homogenized in chloroform/methanol solution, subjected to the extraction of the lipid layer with Triton X-100/chloroform solution, and then resuspended in water. The hepatic TG level was measured using TG-S assay kit (Asan Pharm. Co., Ltd., Seoul, Republic of Korea) according to the manufacturer’s instructions.

### 2.7. Quantitative PCR

Total RNA was extracted with TRIzol^®^ (Thermo Fisher Scientific, Waltham, MA, USA) and reverse-transcribed to complementary DNA using GoScript™ Reverse Transcriptase (Promega, Madison, WI, USA) [19]. Quantitative real-time PCR was performed by using GoTaq^®^ qPCR master mix (Promega) on an ABI StepOnePlus™ fast real-time PCR system (Applied Biosystems, Foster City, CA, USA). Quantification of gene transcripts for acetyl-CoA carboxylase (ACC), acyl-CoA oxidase 1 (Acox1), ATP citrate lyase (ACL), carnitine palmitoyltransferase 1 (CPT1), citrate synthase, diacylglycerol acyltransferase 1 (DGAT1), fatty acid synthase (FAS), hormone-sensitive lipase (HSL), medium-chain acyl-CoA dehydrogenase (mCAD), peroxisome proliferator-activated receptor α (PPARα), PPARγ, PPARγ coactivator 1α (PGC1α), sterol regulatory element-binding protein 1 (SREBP1c), and uncoupling protein 1 (UCP1) was performed using gene-specific primers. Primer sequences are available upon reasonable request. The results were normalized to the expression of acidic ribosomal phosphoprotein (Arbp) using the ΔΔCt method and presented as means ± SD.

### 2.8. Immunoblotting

Immunoblotting was performed as previously explained [19]. Primary antibodies against total AMPK, phospho-AMPK (Thr172), and GAPDH (Cell Signaling Technology, Beverly, MA, USA) were used, with HRP-conjugated anti-rabbit IgG as their secondary antibody (Cell Signaling Technology). 

### 2.9. Cecal Microbiota Analysis and Short-Chain Fatty Acid Measurement

Gut microbial metagenome analysis was performed as described previously [20]. To measure the short-chain fatty acid level, cecal contents were mixed with extraction solution, incubated, centrifuged, filtered, and transferred to a gas chromatography vial (Shimadzu, Kyoto, Japan) [20]. A GC-2010A (Shimadzu, Kyoto, Japan) and HP-Innowax Agilent 30 m × 0.32 mm × 0.25 µm GC column (Agilent Technologies Inc., Santa Clara, CA, USA) were used for detection with N_2_ gas as carrier. Then, 1 μL of sample was injected at 260 °C and detected by a flame ionization detector (FID). The column temperature was increased from 100 °C up to 180 °C at a rate of 25 °C/min. Volatile free acid standard mix (Sigma-Aldrich) was used as the analytical standard for C2 through C5.

### 2.10. Statistical Analyses

The experimental results are presented as means ± SD. Statistical analyses were performed using GraphPad Prism version 9.3.1 (GraphPad, La Jolla, CA, USA). Statistical significance was tested using a two-tailed Student’s *t*-test or one-way analysis of variance (ANOVA), as indicated in each separate experiment, with Dunnett’s multiple comparison test with α = 0.05. *p* values < 0.05 were considered statistically significant.

## 3. Results

### 3.1. GTB1™ Treatment Alleviates High-Fat-Diet-Induced Metabolic Dysfunction in HFD-Fed Mice

**Figure 1:**
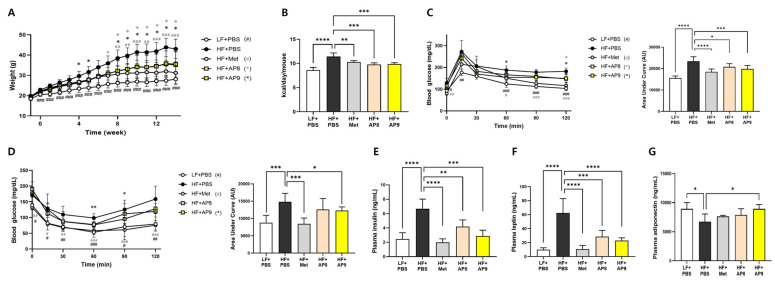
GTB1™ treatment alleviates high-fat-diet-induced metabolic dysfunction in mice. (**A**) Body weight changes for 15 weeks of HF feeding with Lp. plantarum APsulloc 331261 GTB1™ treatment for latter 14 weeks (*n* = 8). (**B**) Average daily calorie intake for 14 weeks of GTB1™ treatment. Glucose tolerance test (**C**) and insulin tolerance test (**D**) after 13 weeks of GTB1™ treatment and the area under curve (*n* = 7~8). Serum concentrations of insulin (**E**), leptin (**F**), and adiponectin (**G**) quantified by ELISA (*n* = 6). Data show mean ± SD. Statistical significance was analyzed using repeated measures (**A**,**C**,**D**) or ordinary one-way ANOVA with Dunnett’s multiple comparison test. * *p* < 0.05, ** *p* < 0.01, *** *p* < 0.001, **** *p* < 0.0001 compared to the HF + PBS group. # *p* < 0.05, ## *p* < 0.01, ### *p* < 0.001 compared to the HF + PBS group. LF, low-fat diet; HF, high-fat diet; PBS, phosphate-buffered saline; Met, metformin; AP8, GTB1™ low dose (1 × 10^8^ CFU/day/mouse); AP9, GTB1™ high dose (1 × 10^9^ CFU/day/mouse).

The administration of *Lp. plantarum* APsulloc 331261 GTB1™ significantly reduced HF-feeding-induced increase in body weight gain (Figure 1A) and calorie intake (Figure 1B) in both low- and high-dose-treated groups. In the oral glucose tolerance test, the blood glucose concentration was significantly lower at 120 min in the low-dose treated group compared to the HFD-fed group, and at 60 and 120 min in the high-dose treated group (Figure 1C). In the insulin tolerance test, both low- and high-dose-GTB1™-treated mice showed a decreased blood glucose concentration (Figure 1D); however, only for the high-dose treatment was this statistically significant. In addition, plasma insulin and leptin levels were elevated by HF feeding, and these levels were lowered by probiotic treatment (Figure 1E,F). On the other hand, the high-dose-GTB1™-treated group presented a significantly increased circulating adiponectin concentration (Figure 1G), and the phosphorylation level of AMP-activated protein kinase (AMPK), which is a downstream target of adiponectin, was also increased in the skeletal muscle (Figure S1). Together, these results indicate that GTB1™ attenuates HFD-induced body weight gain, glucose intolerance, and insulin resistance.

### 3.2. GTB1™ Treatment Suppresses Tissue Adiposity and Improves Plasma Lipid Profile in HFD-Fed Mice

**Figure 2:**
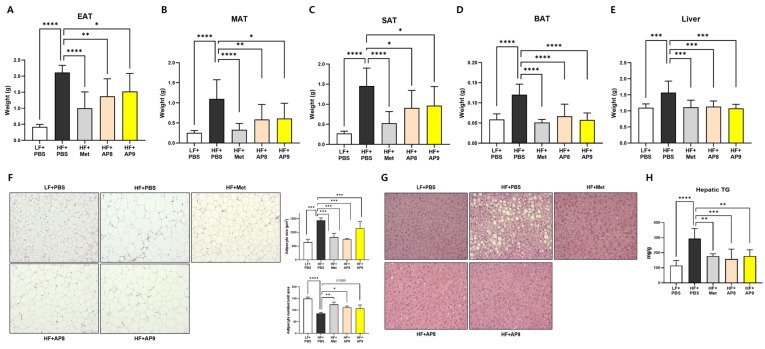
GTB1™ treatment suppresses tissue adiposity in HFD-fed mice. Tissue weight changes in EAT (**A**), MAT (**B**), SAT (**C**), BAT (**D**), and the liver (**E**) after 14 weeks of *Lp. plantarum* APsulloc 331261 GTB1™ treatment (*n* = 8). Changes in adipocyte size and cell population in EAT (**F**) and hepatic adiposity (**G**) after GTB1™ treatment (*n* = 3). Shown here are representative photomicrographs of each tissue section stained with hematoxylin and eosin (100×). (**H**) Effect of GTB1™ treatment on hepatic TG accumulation (*n* = 8). Data show mean ± SD. Statistical significance between experimental groups was analyzed using ordinary one-way ANOVA with Dunnett’s multiple comparison test. * *p* < 0.05, ** *p* < 0.01, *** *p* < 0.001, **** *p* < 0.0001 compared to the HF + PBS group. EAT, epididymal adipose tissue; MAT, mesenteric adipose tissue; SAT, subcutaneous inguinal adipose tissue; BAT, brown interscapular adipose tissue; TG, triglyceride; LF, low-fat diet; HF, high-fat diet; PBS, phosphate-buffered saline; Met, metformin; AP8, GTB1™ low dose (1 × 10^8^ CFU/day/mouse); AP9, GTB1™ high dose (1 × 10^9^ CFU/day/mouse).

The attenuation of HFD-induced weight gain in GTB1™-treated mice was observed parallel to a significant reduction in tissue weight, including in EAT, MAT, SAT, BAT, and the liver (Figure 2A–E). Histological analysis of EAT showed a significant decrease in adipocyte size and an increase in the numbers of adipocytes by GTB1™ treatment (Figure 2F). Also, the histological examination of the liver presented a meaningfully reduced fat deposition and a concomitant decrease in hepatic TG accumulation in both low- and high-dose probiotic-treated groups (Figure 2G,H). 

**Figure 3:**
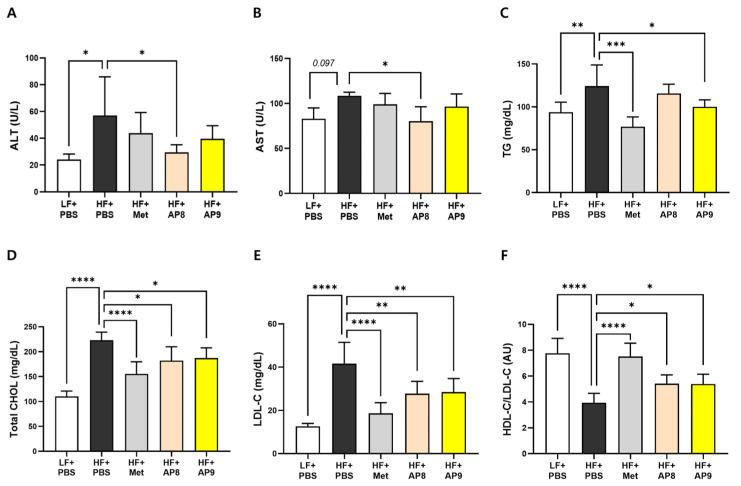
GTB1™ treatment improves plasma lipid profiles in HFD-fed mice. Plasma levels of ALT (**A**), AST (**B**), triglycerides (**C**), total cholesterol (**D**), LDL-cholesterol (**E**), and the ratio of HDL-cholesterol over LDL-cholesterol (**F**) after 14 weeks of *Lp. plantarum* APsulloc 331261 GTB1™ treatment. Data show mean ± SD. Statistical significance between experimental groups was analyzed using ordinary one-way ANOVA with Dunnett’s multiple comparison test. * *p* < 0.05, ** *p* < 0.01, *** *p* < 0.001, **** *p* < 0.0001 compared to the HF + PBS group. ALT, alanine transaminase; AST, aspartate aminotransferase; TG, triglycerides; Total CHOL, total cholesterol; LDL-C, low-density lipoprotein-cholesterol; HDL-C, high-density lipoprotein-cholesterol; LF, low-fat diet; HF, high-fat diet; PBS, phosphate-buffered saline; Met, metformin; AP8, GTB1™ low dose (1 × 10^8^ CFU/day/mouse); AP9, GTB1™ high dose (1 × 10^9^ CFU/day/mouse).

Circulating levels of ALT and AST were significantly decreased in the low-dose-GTB1™-treated group compared to the HFD-fed controls (Figure 3A,B). In addition, plasma lipid profiles showed a significant decrease in triglycerides, total cholesterol, and LDL-cholesterol, and an increase in HDL-cholesterol/LDL-cholesterol ratio with both low- and high-dose GTB1™ treatment (Figure 3C–F). These data indicate that the administration of GTB1™ alleviates HFD-induced tissue adiposity and dysregulated circulating lipid levels.

### 3.3. GTB1™ Treatment Ameliorates Dysregulated Lipid Metabolism in Adipose Tissues of HFD-Fed Mice

**Figure 4:**
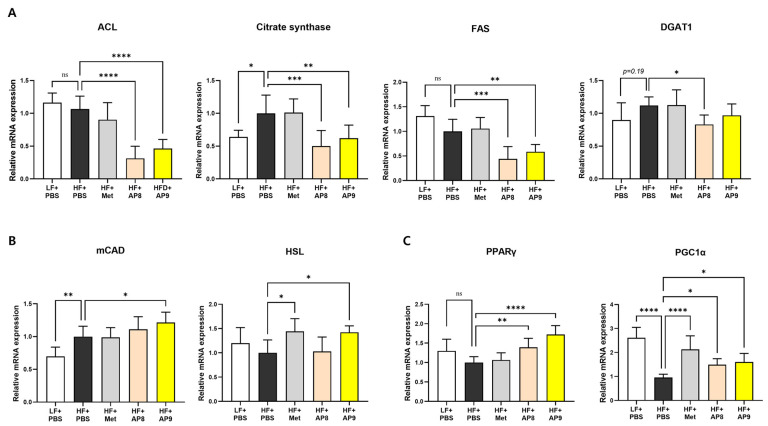
GTB1™ treatment alters the expression of lipid-metabolic genes in HFD-fed mice. Effect of 14 weeks’ treatment of GTB1™ on gene expression of lipogenesis (**A**), lipolysis (**B**), and lipid metabolism regulators (**C**) in EAT. All genes were normalized to the expression of Arbp (*n* = 8). Data show means ± SD. Statistical significance between experimental groups was analyzed using ordinary one-way ANOVA with Dunnett’s multiple comparison test. * *p* < 0.05, ** *p* < 0.01, *** *p* < 0.001, **** *p* < 0.0001 compared to the HF + PBS group. EAT, epididymal adipose tissue; LF, low-fat diet; HF, high-fat diet; PBS, phosphate-buffered saline; Met, metformin; AP8, GTB1™ low dose (1 ×10^8^ CFU/day/mouse); AP9, GTB1™ high dose (1 × 10^9^ CFU/day/mouse); ns: not significant.

**Figure 5:**
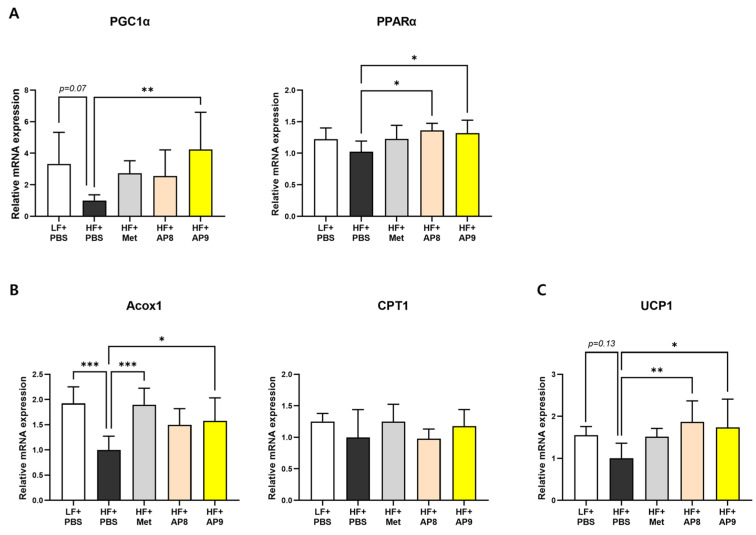
GTB1™ treatment modulates thermogenic gene expression in BAT of HFD-fed mice. Effect of 14 weeks’ treatment of GTB1™ on thermogenic-regulatory (**A**), lipid-oxidative (**B**), and thermogenic (**C**) gene expression of BAT. All genes were normalized to the expression of Arbp (*n* = 8). Data show mean ± SD. Statistical significance was analyzed using ordinary one-way ANOVA with Dunnett’s multiple comparison test. * *p* < 0.05, ** *p* < 0.01, *** *p* < 0.001 compared to the HF + PBS group. BAT, brown interscapular adipose tissue; LF, low-fat diet; HF, high-fat diet; PBS, phosphate-buffered saline; Met, metformin; AP8, GTB1™ low dose (1 × 10^8^ CFU/day/mouse); AP9, GTB1™ high dose (1 × 10^9^ CFU/day/mouse).

To examine how GTB1™ treatment reduced adiposity in metabolic tissues, changes in the expression of genes related to lipid metabolism were analyzed in EAT, BAT, and the liver. In EAT, the expression levels of lipogenic genes including ACL, Citrate synthase, FAS, and DGAT1 were lower in GTB1™-treated mice than HFD-fed controls (Figure 4A), whereas the expression of the lipolytic genes, mCAD and HSL, and the master regulatory genes of lipid metabolism, PPARγ and PGC1α, were significantly increased by GTB1™ treatment (Figure 4B,C). The mRNA levels of PGC1α, PPARα, and thermogenic genes including Acox1 and UCP1 were markedly increased in the BAT of the GTB1™-treated group compared to the HFD-fed controls (Figure 5). On the other hand, there was no significant change in the gene expression in the hepatic lipid metabolism by GTB1™ treatment (Figure S2).

### 3.4. GTB1™ Treatment Augments Short-Chain Fatty Acid Production and Modulates Microbiota Composition in HFD-Fed Mice

**Figure 6:**
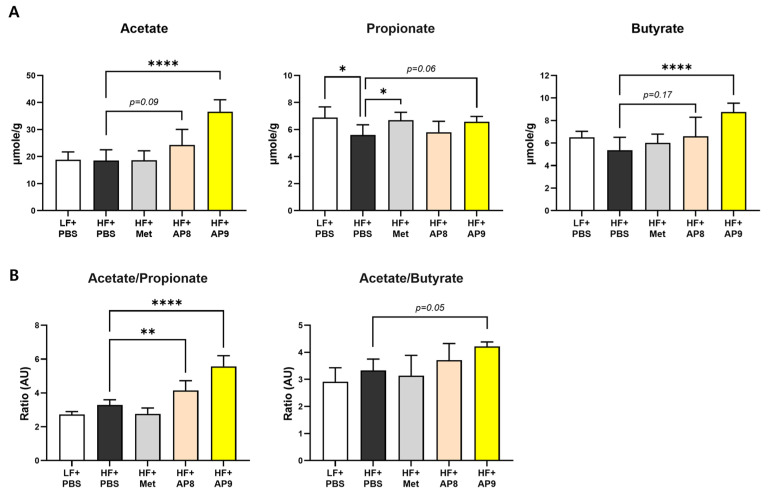
GTB1™ treatment augments short-chain fatty acid production in HFD-fed mice. Changes in short-chain fatty acid concentration including acetate, propionate, and butyrate (**A**), and the ratio of acetate/propionate and acetate/butyrate (**B**) in the cecal contents after 14 weeks of GTB1™ treatment (*n* = 6~8). Data show mean ± SD. Statistical significance was analyzed using ordinary one-way ANOVA with Dunnett’s multiple comparison test. * *p* < 0.05, ** *p* < 0.01, **** *p* < 0.0001 compared to the HF + PBS group. LF, low-fat diet; HF, high-fat diet; PBS, phosphate-buffered saline; Met, metformin; AP8, GTB1™ low dose (1 × 10^8^ CFU/day/mouse); AP9, GTB1™ high dose (1 × 10^9^ CFU/day/mouse).

**Figure 7:**
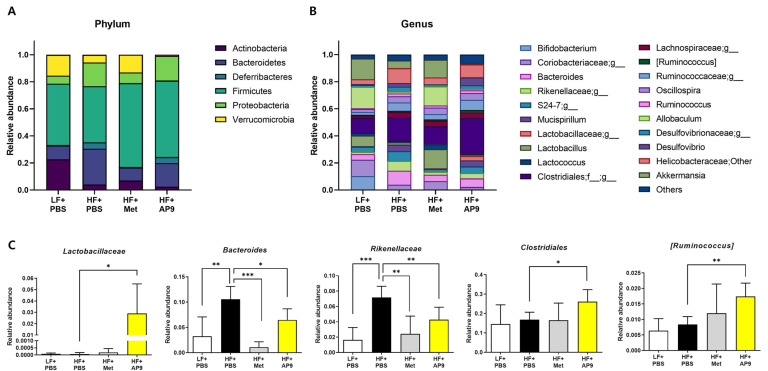
GTB1™ treatment modulates microbiota composition in HFD-fed mice. Changes in cecal microbiota composition at the levels of phylum (**A**) and genus (**B**). (**C**) Relative abundance of different taxonomic groups by GTB1™ treatment. Data show mean ± SD (*n* = 6~8). Statistical significance between experimental groups was analyzed using ordinary one-way ANOVA with Dunnett’s multiple comparison test. * *p* < 0.05, ** *p* < 0.01, *** *p* < 0.001 compared to the HF + PBS group. LF, low-fat diet; HF, high-fat diet; PBS, phosphate-buffered saline; Met, metformin; AP9, GTB1™ high dose (1 × 10^9^ CFU/day/mouse).

To determine whether the beneficial metabolic effects of GTB1™ were related to alterations to HFD-induced microbiota dysbiosis, the cecal short-chain fatty acids and profiles of cecal microbiota were analyzed. The amount of short-chain fatty acids including acetate, propionate, and butyrate was increased dose-dependently in the GTB1™-treated group, and the ratio of acetate/propionate and acetate/butyrate was increased (Figure 6). Principal component analysis (PCA) of β-diversity present in the cecal bacterial community showed a significant shift in PC3 (Figure S3), and the taxonomic composition was also altered by GTB1™ treatment (Figure 7A,B). Specifically, the relative abundance of Bacteroides and *Rikenellaceae* was significantly lower in the GTB1™-treated group, while the abundance of *Lactobacillaceae*, Clostridiales, and [*Ruminoccus*] was augmented (Figure 7C). Taken together, these data indicate that the consumption of GTB1™ was able to improve microbiota dysbiosis, which could contribute to augmentations in HFD-induced metabolic dysfunctions.

## 4. Discussion 

An imbalance between energy consumption and expenditure causes overnutrition status, which results in dysregulated metabolic status, including excessive adiposity, insulin resistance, hyperlipidemia, and related diseases [21]. Probiotics has been proposed as a potent therapeutic strategy for metabolic disorders by modulating the intestinal microbiota composition, producing host-effective bioactive molecules, and regulating immune responses [9,10,11]. The metabolic improving effect of different probiotic strains on metabolic dysfunction has been constantly reported. A probiotic mixture of *B. animalis* subsp. *lactis* and *Lb. gasseri* showed reduced adiposity contributed by modulating intestinal Farnesoid X receptor signaling [22]. Treatment with *Limosilactobacillus fermentum*, *Lacticaseibacillus casei*, or *Lactococcus lactis* showed lowered adiposity and plasma glucose levels with attenuated pro-inflammatory cytokines in a strain-dependent manner [23]. *Akkermansia muciniphila*, a representative of next-generation probiotics, has been reported to prevent HFD-induced hepatic inflammation by regulating the TLR2-activated γδT17 cell population and macrophage polarization [24]. In the present study, a probiotic strain, *Lp. plantarum* APsulloc 331261 (GTB1™), was examined to test its protective effect against metabolic dysregulation and to investigate its underlying mechanisms. The strain was first isolated from green tea leaves, identified as a safe *Lp. plantarum*, and assessed for probiotic features, including high survivability in the simulated gastrointestinal (GI) environment and a high adhesion rate to human intestinal epithelial cells [17]. Furthermore, it was clearly confirmed that the strain is distinguishable from the other *Lp. plantarum* strains by comparing the whole genome sequence. 

The *Lp. plantarum* species was originally derived from a plant; however, it possesses cassettes of carbohydrate-utilizing genes, which allow the strain to adapt to various ecological conditions, including the GI tract [25]. In the HFD-induced obese mouse model, GTB1™ treatment substantially reduced body weight gain, daily calorie intake, and glucose and insulin intolerance (Figure 1A–D). Significant improvement was also observed in the circulating lipid profiles, including TG and cholesterol composition, of GTB1™-treated mice (Figure 3C–F). Taken together with the attenuated tissue adiposity and hepatic TG accumulation (Figure 2), this suggests that supplementation with GTB1™ could alleviate lipid overload and thus improve hyperlipidemia induced by HFD feeding. Plasma biochemistry analysis also revealed significant decreases in ALT and AST levels, respectively, in GTB1™ low-dose-treated HFD-fed mice and a decreasing tendency in the high-dose-treated group (*p* = 0.10 for ALT, *p* = 0.19 for AST). Unexpectedly, the improvement in circulating ALT and AST did not show dose dependency, which indicates that a more sophisticated dose selection should be carried out to minimize any potential side effects.

Plasma analyses revealed recovery from the HFD-induced increase in insulin and leptin levels by GTB1™ treatment dose-dependently (Figure 1E,F), indicating that resistance to insulin and leptin signaling was partially resolved. In addition, there was recovery from the suppressed production of adiponectin in the GTB1™-treated group, followed by the promotion of AMPK phosphorylation at Thr 172 in the skeletal muscle tissue (Figure 1G and Figure S1). Adiponectin is a potent organokine that is exclusively secreted from adipose tissues and exerts beneficial effects, such as improvements in insulin sensitivity, glucose/lipid metabolism, and chronic inflammation in peripheral tissues, and its intracellular signaling is known to be mediated by AMPK activation [26,27]. Taken together, our results suggest that the protective effect of GTB™ treatment in metabolic dysregulation could be mediated by enhanced adiponectin production.

Adipocyte hypertrophy is a pathogenic condition that results from an overaccumulation of lipid beyond the tissue buffering capacity, eliciting metabolic stress, such as tissue hypoxia, endoplasmic reticulum and oxidative stress, low-chronic inflammation, and ectopic fat deposition [28,29]. In our study, the administration of GTB1™ suppressed both white and brown adipose tissue expansion (Figure 2A–D), accompanied by significantly reduced adipocyte hypertrophy and moderately enhanced hyperplasia (Figure 2F), indicating a convalesced adipose tissue function. This was further corroborated by the changes in the gene expression patterns of EAT and BAT. GTB1™ treatment suppressed lipogenic gene expression, whereas the expression of genes related to lipolysis and master regulators of lipid metabolism, PPARγ and its coactivator PGC1α, was augmented in EAT (Figure 4). PPARγ was first described as a factor induced during adipocyte differentiation and is known widely for its role in the regulation of adipogenesis and lipogenic pathways [30]. It also plays a crucial role in improving glucose uptake by enhancing the expression of glucose transporters, and in regulating the expression of adipokines, such as adiponectin, resistin, and leptin [31], which further convey insulin-sensitizing signals; thus, numerous anti-diabetic drugs such as thiazolidinediones (TZDs) were developed to stimulate PPARγ activity [30]. PGC1α is also considered a pivotal factor for energy metabolism [32]. Concomitant with PPAR expression, PGC1α is known to regulate the whole process of the mitochondrial life cycle and its sequential events, such as the trans-differentiation of white adipocytes into beige adipocytes, facilitating adipose tissue browning and the stimulation of thermogenic activity in brown adipocytes [33,34]. In our study, GTB1™ treatment substantially upregulated the expression of PGC1α and PPARα and their downstream targets, including Acox1 and UCP1 (Figure 5). Interestingly, the upregulation of the PPAR/PGC1α pathway was only observed in adipose tissues, not in the liver (Figure S2); however, GTB1™-treated mice showed improvements in HFD-induced hepatomegaly, hepatic steatosis represented by TG level (Figure 2E,G,H), and circulating lipid profiles (Figure 3). In summary, supplementation with GTB1™ could relieve energy-metabolic stress by modulating PPAR/PGC1α signaling in adipose tissues, which contributes to improvements to dysregulated metabolism features, including excessive adiposity, insulin resistance, hepatic steatosis, and hyperlipidemia. 

Nutritional imbalance causes dysbiosis in the gut microbiota, and probiotic consumption could repair an abnormal energy metabolism by modulating the microbiota composition and its byproducts [35]. Many studies have reported that the intestinal microbiota of obese individuals/animals has a less diverse composition compared to healthy controls, as well as compromised colonic fermentation and SCFA synthesis [36]. Additionally, SCFAs produced from the microbiota serve as an energy source for enterocytes and major signaling molecules in peripheral tissues by stimulating G-protein-coupled receptors (GPR 41, 43, 109), leading to improvements to metabolic disorders by enhancing the activities of intracellular mediators, such as AMPK and SIRT1 [37]. Supplementation with SCFAs also switched the PPARγ activity from lipogenesis to lipid oxidation by stimulating the UCP2-AMPK-ACC pathway [38]. In this study, cecal concentrations of acetate, propionate, and butyrate and their ratio were increased dose-dependently by GTB1™ treatment (Figure 6). The diversity of the cecal microbiota showed significant shifts in β-diversity (Figure S3) and taxonomic composition (Figure 7A,B) by GTB1™ administration. Notably, the population of *Lactobacillaceae*, Clostridiales, and [*Ruminococcus*], which are SCFA-producing groups, was increased in the GTB1™-treated group (Figure 7C). These data suggest that the administration of GTB1™ to the high-fat-diet-induced obese mouse amended intestinal microbiota dysbiosis, particularly by potentiating SCFA production. 

In summary, our present data demonstrate that *Lp. plantarum* APsulloc 331261 (GTB1™) is a promising candidate strain for preventing HFD-induced metabolic pathologies through restoring adipose tissue function, intestinal microbiota dysbiosis, and SCFA production. These findings conclusively provide evidence to suggest that GTB1™ is a potential agent for intervention in metabolic disorders. Further study should be carried out to identify effective components working as PPAR agonists, such as exopolysaccharides, S-layer proteins, or secondary metabolites of GTB1™. Also, investigation into the host cellular response to the altered intestinal microbiota composition, including changes in mucosal immune status through short-chain fatty acids, would provide a comprehensive understanding of the use of GTB1™ against metabolic disorders.

## Data Availability

The original contributions presented in the study are included in the article, further inquiries can be directed to the corresponding authors.

## Supplementary Materials

The following supporting information can be downloaded at: https://www.mdpi.com/article/10.3390/foods13142227/s1, Figure S1: GTB1™ treatment enhances AMPK phosphorylation in skeletal muscle tissue of HFD-fed mice; Figure S2: There is no significant alteration to hepatic lipid metabolism by GTB1™ treatment in HFD-fed mice; Figure S3: The alterations in the diversity of cecal microbiota by GTB1™ treatment.
