# Supplementary material for: Comprehensive Amelioration of Metabolic Dysfunction through Administration of Lactiplantibacillus plantarum APsulloc 331261 (GTB1™) in High-Fat-Diet-Fed Mice
Source: Foods. 2024 Jul 16;13(14):2227. doi: 10.3390/foods13142227 (PMC11276112; doi:10.3390/foods13142227)
Supplement: Supplementary file 1 [file foods-13-02227-s001.zip › foods-3075563-supplementary.pdf]

Comprehensive Amelioration of Metabolic Dysfunction through Administration of *Lactiplantibacillus plantarum* APsulloc 331261 (GTB1™) in High-Fat-Diet-Fed Mice

Bobae Kim[1,2,†], Yuri Lee[1,2,†], Chungho Lee[1], Eun Sung Jung[3], Hyeji Kang[1,4,*] and Wilhelm H. Holzapfel[1,2,*]

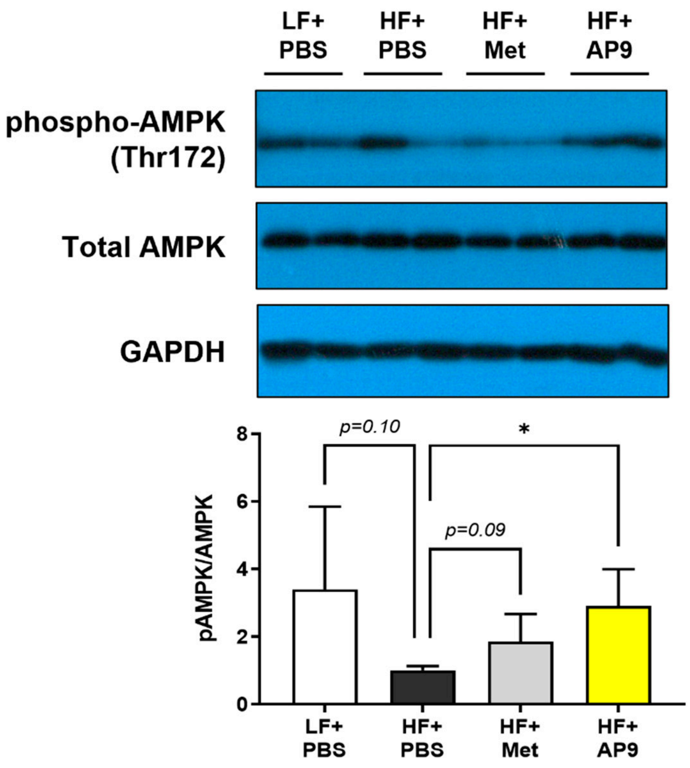

Supplementary Figure S1. GTB1™ treatment enhances AMPK phosphorylation in skeletal muscle tissue of HFD-fed mice. Changes of AMPK phosphorylation level in quadriceps tissue after 14 weeks of GTB1™ treatment. GAPDH was used as a loading control. Data present mean ± SD. Statistical significance between experimental groups were analyzed using ordinary one-way ANOVA with Dunnett's multiple comparison test. * p < 0.05 compared to the HF+PBS group. LF: low-fat diet, HF: high-fat diet, PBS: phosphate buffered saline, Met: metformin, AP9: GTB1™ high dose (1×10^9 CFU/day/mouse).

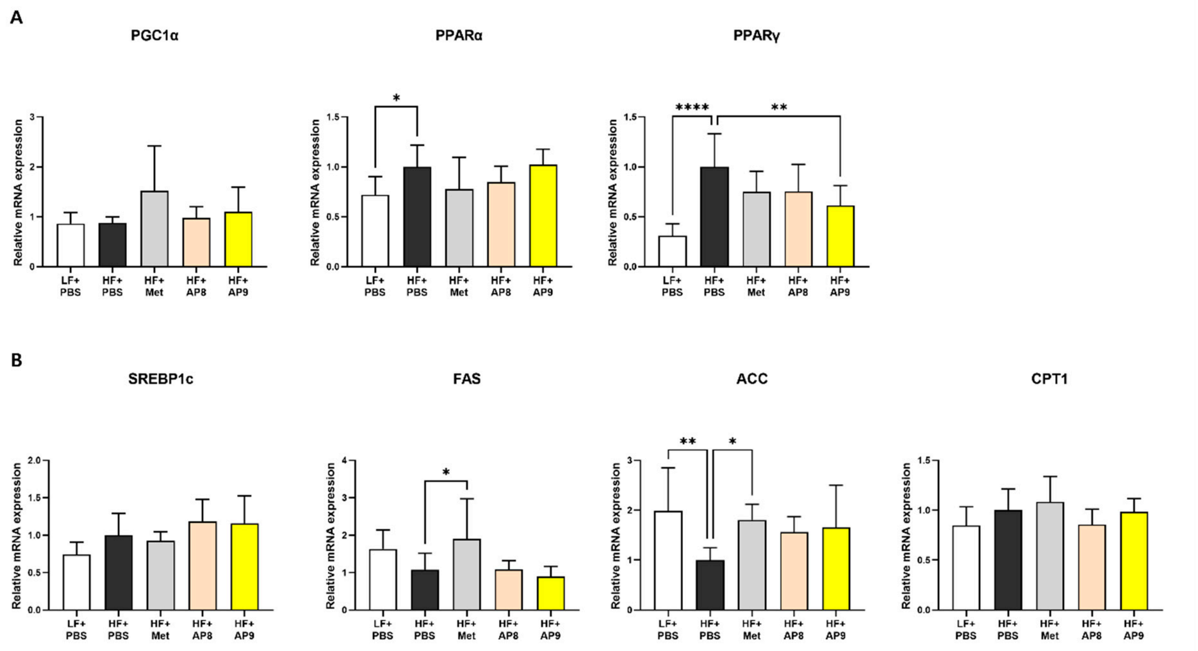

Supplementary Figure S2. There is no significant alteration to hepatic lipid metabolism by GTB1™ treatment in HFD-fed mice. Hepatic mRNA levels of genes related to the transcriptional regulation (**A**), and lipid synthesis (**B**) in lipid metabolism after 14 weeks of GTB1™ treatment. All genes were normalized to the expression of Arbp ($n = 8$). Data present mean ± SD. Statistical significance between experimental groups were analyzed using ordinary one-way ANOVA with Dunnett's multiple comparison test. $^{*}p < 0.05$, $^{**}p < 0.01$, $^{****}p < 0.0001$ compared to the HF+PBS group. LF: low-fat diet, HF: high-fat diet, PBS: phosphate buffered saline, Met: metformin, AP8: GTB1™ low dose (1×10^8 CFU/day/mouse), AP9: GTB1™ high dose (1×10^9 CFU/day/mouse).

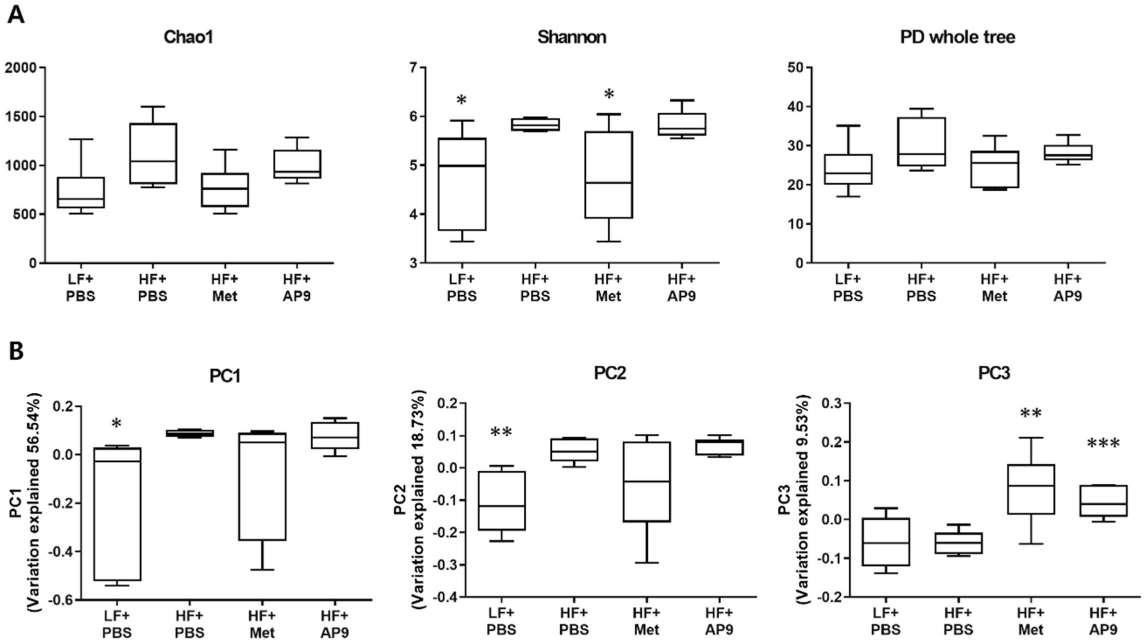

Supplementary Figure S3. The alterations in the diversity of cecal microbiota by GTB1™ treatment. Microbial analyses on the cecal contents. (**A**) α-diversity, (**B**) β-diversity. * $p < 0.05$, ** $p < 0.01$, *** $p < 0.001$ compared to the HF + PBS group.
